# Electrogastrography in Autonomous Vehicles—An Objective Method for Assessment of Motion Sickness in Simulated Driving Environments

**DOI:** 10.3390/s21020550

**Published:** 2021-01-14

**Authors:** Timotej Gruden, Nenad B. Popović, Kristina Stojmenova, Grega Jakus, Nadica Miljković, Sašo Tomažič, Jaka Sodnik

**Affiliations:** 1 Faculty of Electrical Engineering, University of Ljubljana, Tržaška c. 25, 1000 Ljubljana, Slovenia; timotej.gruden@fe.uni-lj.si (T.G.); kristina.stojmenova@fe.uni-lj.si (K.S.); grega.jakus@fe.uni-lj.si (G.J.); saso.tomazic@fe.uni-lj.si (S.T.); 2 School of Electrical Engineering, University of Belgrade, B. kralja Aleksandra 73, 11000 Belgrade, Serbia; nenad.pop92@gmail.com (N.B.P.); nadica.miljkovic@etf.rs (N.M.)

**Keywords:** electrogastrography, autonomous vehicle, motion sickness, driving simulator, virtual reality

## Abstract

Autonomous vehicles are expected to take complete control of the driving process, enabling the former drivers to act as passengers only. This could lead to increased sickness as they can be engaged in tasks other than driving. Adopting different sickness mitigation techniques gives us unique types of motion sickness in autonomous vehicles to be studied. In this paper, we report on a study where we explored the possibilities of assessing motion sickness with electrogastrography (EGG), a non-invasive method used to measure the myoelectric activity of the stomach, and its potential usage in autonomous vehicles (AVs). The study was conducted in a high-fidelity driving simulator with a virtual reality (VR) headset. There separate EGG measurements were performed: before, during and after the driving AV simulation video in VR. During the driving, the participants encountered two driving environments: a straight and less dynamic highway road and a highly dynamic and curvy countryside road. The EGG signal was recorded with a proprietary 3-channel recording device and Ag/AgCl cutaneous electrodes. In addition, participants were asked to signalize whenever they felt uncomfortable and nauseated by pressing a special button. After the drive they completed also the Simulator Sickness Questionnaire (SSQ) and reported on their overall subjective perception of sickness symptoms. The EGG results showed a significant increase of the dominant frequency (DF) and the percentage of the high power spectrum density (FSD) as well as a significant decrease of the power spectrum density Crest factor (CF) during the AV simulation. The vast majority of participants reported nausea during more dynamic conditions, accompanied by an increase in the amplitude and the RMS value of EGG. Reported nausea occurred simultaneously with the increase in EGG amplitude. Based on the results, we conclude that EGG could be used for assessment of motion sickness in autonomous vehicles. DF, CF and FSD can be used as overall sickness indicators, while the relative increase in amplitude of EGG signal and duration of that increase can be used as short-term sickness indicators where the driving environment may affect the driver.

## 1. Introduction

Current research foresees significant improvement in safety, fuel consumption, time efficiency and driver comfort with the introduction of autonomous vehicles (AVs). With the vehicle taking complete control of the driving process, the drivers’ role is expected to change completely, enabling them to work or entertain themselves throughout the journey. However, it has been shown that engaging in tasks such as reading [1] or performing visual search tasks [2] that deprive passengers of a clear view of the external moving environment, can lead to increased motion sickness (MS), introducing a new challenge for autonomous vehicles’ infotainment designs [3,4,5,6]. Iskander et al. have written an extensive review on the new “autonomous car sickness” (i.e., motion sickness, perceived in autonomous vehicles) in comparison to classic “car sickness” [4], expressing the need to separately study MS in AVs. Since the occurrence and severity of MS in AVs are expected to increase [3,7], the general acceptance of AVs could be questionable without proper countermeasures [8]. Reduced comfort offered by the AVs due to MS can overshadow their other benefits which are more important for the society (reduced fuel consumption, security, car sharing possibilities, etc.). One of the suggested approaches to MS symptoms mitigation in AVs is a smoother lateral acceleration than a human driver may perform [9,10], possibly leading to different (milder) sickness in comparison to passenger sickness in human-driven vehicles. Finding a real-time method for assessment of MS or its symptoms in autonomous vehicles could be therefore useful for early detection and development of in-vehicle infotainment concepts and driving algorithms that could further reduce or minimize MS symptoms.

According to the sensory conflict theory, passive movement in vehicles can create a conflict between vestibular and visual inputs (a mismatch of senses) which causes nausea attributed to MS [11]. The main symptoms of MS include (but are not limited to) nausea, vomiting, sweating, eye-strain, difficulty focusing, headaches, oculomotor disturbances, disorientation, dizziness and vertigo [12,13,14,15]. They can be assessed either subjectively as perceived by drivers or via their physiological correlates. The most commonly used subjective methods for assessing MS in vehicles include the Motion Sickness Questionnaire (MSQ) [16,17] or its derivative—Simulator Sickness Questionnaire (SSQ) [18] for detailed overall evaluation of MS, Fast Motion Sickness Scale (FMS) [19] for faster multiple evaluations during the study trials and Motion Sickness Susceptibility Questionnaire (MSSQ) [20] or Georgia Tech Simulator Sickness Screening Protocol Paper (GTSSSP) [21] for screening drivers’ susceptibility to MS. Some researchers also use separate Likert scales to assess different aspects of MS [5]. Subjective MS effects identified with subjective assessments are usually collected post-trial and provide an overall score. This does not always enable identification on specific events that lead to MS, which is very important information when trying to eliminate or at least minimize it. It is therefore important to investigate methods for real time and continuous assessment of MS, which can be time and event correlated. As subjective measurements could sometimes be biased, there is also an ongoing need in the research community for the development of objective measurement methods [4,5]. Despite low overall success, correlations of drivers’ physiological signals with the symptoms of MS have been established through heart rate variability [22,23,24], body temperature [25], skin conductance [25,26,27] and electrogastrogram [27,28]. Among the research, the most often reported and widely studied MS symptom is nausea [4,5,11,25,29,30]. Since nausea and vomiting come from gastrointestinal distress [12,18], there is a reasonably high probability for successful detection of MS with techniques that are primarily used to monitor the gastrointestinal tract [31,32].

A noninvasive measurement method called electrogastrography (EGG) seems very promising for the task [31,33,34]. It is the technique for measuring gastric myoelectrical activity in stomach smooth muscle cells from surface electrodes, positioned on the abdominal surface of the body [35], which was first used and described by Alvarez in 1922 [36]. Medical doctors and other scientists can gather a lot of useful information about the gastric activity from observing the EGG recordings [37], e.g., detect tachygastria (increased dominant frequency) or bradygastria (decreased dominant frequency) from spectral components, functional dyspepsia [38] and abnormal gastric emptying [39,40]. EGG has also been successfully used in other research fields, such as personalized modeling [41], lie detection [42] and user-interface design [43]. Modern measuring techniques include swallowing an active ingestible capsule (called enhanced electrogastrography, EEGG) [44] or wearing a high-resolution torso-tank with many electrodes [45,46].

The myoelectrical activity of stomach muscles (EGG) consists of two components, “*slow waves*” and “*spike potentials*”, of which researchers mainly find the first one—slow waves—easier to reliably measure [35,46,47]. Slow waves are often referred to as electrical activity that controls gastric contractions. It was shown that not all slow waves are accompanied with a gastric contraction, but a slow wave is always present when a contraction occurs. Therefore it is believed that only a slow wave with amplitude above a certain threshold causes a gastric contraction [35,48]. A slow wave is a sinusoidal signal with dominant frequency (DF) of about 3 cpm (2–4 cpm) and amplitude of 100–500 µV in healthy subjects. The phenomenon of DF not in normal range is called gastric dysrhythmia. It is expected for the DF of slow wave and its power to increase (shift to tachygastric range) while experiencing sickness [28,31,49,50].

Over the past 30 years, EGG has been widely used as an objective measurement of nausea and in developing anti-MS drugs [31]. However, making strong conclusions from examining EGG signals for motion or simulation sickness detection is still questionable by some researchers and the results of EGG studies should therefore be interpreted with caution [32,51]. While researching EGG in motion sickness, Cheung and Vaitkus reported that inherent inter-subject variability makes it difficult to consider EGG a reliable indicator of MS [31]. After a few years, Tokumaru et al. reported two different kinds of tachygastria due to MS—one with and one without a change in normal slow waves [50]. Himi et al. measured nausea with EGG on people, watching an irregularly oscillating video, and discussed the role of autonomic nervous system in nausea [52]. Their results demonstrated the defensive reactions of sympathetic nervous system against nausea. Lien et al. suggested another countermeasure for MS—ginger [53]. They reported it reduces nausea and tachygastric activity. After some years Koch again pointed out gastric dysrhythmias measured with EGG as a potential objective measurement of nausea due to MS [54].

More recent research results showed that EGG is a strong indicator of nausea [35,43,54], and as such has been widely used in numerous situations related to sickness and motion sickness. A recent survey has also revealed that EGG is the third most often used objective measure (EGG, electrodermal activity, electroencephalography and eye-related measurements share the place) of virtual reality (VR) sickness [55]. Dennison et al. found a statistically significant correlation of bradygastric power with disorientation and cybersickness measured with SSQ, while no correlation of SSQ measures with other physiological parameters (heart-rate, electrodermal activity, etc.) could have been established [43]. Vujic et al. have recently begun the construction of wearable EGG device which could be used for nausea detection [56].

There are very few up-to-date studies on assessing motion or simulation sickness with EGG during driving in vehicles and almost none investigating motion sickness with EGG during autonomous driving. Mühlbacher et al. considered EGG for measuring motion sickness during autonomous driving in their methodological recommendations [5]. They concluded it could only show valuable information when drivers are not moving or speaking and therefore the use of EGG in real vehicles could be subject to severe motion artefacts. Miljković et al. on the other hand assessed sickness in virtual environments and suggested that EGG is a promising procedure for cyber sickness and simulator sickness assessment [57]. Popović et al. have constructed their own simple gastric motility device for measuring EGG [58] and used an improved version for assessment of gastric motility in a driving simulator [32]. They concluded that slow waves can be recorded during driving simulation and presented the need to further study the effects of motion and simulation sickness on EGG.

This paper reports on the continuation of the research reported by Popović et al. [32,58]. In the referenced study [32] they demonstrated high potential of using EGG for assessment of simulation sickness in a driving simulator while this research upgrades the study in the following ways:

- it uses the improved (compact printed circuit board) version of the same EGG measuring device;
- it takes place in simulated fully autonomous vehicle (using VR);
- it correlates EGG measurements with subjectively reported sickness (through button presses) and additional physiological responses (galvanic skin response—GSR);
- it investigates also the impact of different driving environments on perceived nausea and sickness

This study therefore proposes the following main research question: “Can EGG be used for detection of motion sickness in a simulated autonomous (self-driving) vehicle?”

Other (secondary) questions include:

- Does driving environment affect the perceived motion sickness?
- Do EGG measurements correlate with subjectively reported nausea onsets?
- Do two different types of self-reported data correlate (questionnaires vs. frequency of reported nausea onsets through button presses)?

The rest of the paper is organized as follows: a detailed description of a conducted user study in driving simulator is presented in the following section; results of EGG recordings when resting, riding in simulated AV and resting again after the ride are presented in Section 3; a brief discussion is presented in Section 4; finally the main conclusions of this study are presented in Section 5.

## 2. Materials and Methods

### 2.1. Study Design

We present a user study with a within-subject design, where we observed test participant’s state before, during and after AV driving simulation, and while driving in different driving environments. The main *independent variable* was the simulation of AV ride {“before”, “during”, “after”}. The intensity of the AV ride was also manipulated through two different driving environments.

The study consisted of three approximately 15-min long trials: (1) baseline measurement prior to driving, (2) driving simulation in AV in the driver’s seat and (3) an “after drive” resting measurement. Trial 2 consisted of a less dynamic drive, which took part on a highway road (Part A) and a more dynamic drive, which took part on a countryside road (Part B).

The following parameters (*dependent variables*) were calculated to assess MS:

- EGG:
  ○ RMS—root mean square value of the signal,
  ○ MF—median frequency of the signal,
  ○ MFM—maximum magnitude of power spectrum density,
  ○ DF—dominant frequency (location of MFM),
  ○ CF—Crest factor of Power Spectrum Density,
  ○ FSD—Percentage of PSD that has higher value than MFM/4,
  ○ amount of time with amplitude increase (Trial 2 only),
  ○ increase in RMS value of the signal segment with an amplitude increase relative to baseline RMS value (Trial 2 only),
- GSR (galvanic skin response):
  ○ mean,
  ○ standard deviation,
- HR (heart rate):
  ○ mean,
  ○ standard deviation,
- Subjective assessment methods:
  ○ number of nausea onsets (by pressing the button, Trial 2 only),
  ○ SSQ nausea score.

Prior to the main experimental autonomous drive simulation, participants completed a test trial to get familiar with the simulation environment and equipment used in the experiment.

### 2.2. Driving Environment

**Figure 1:**
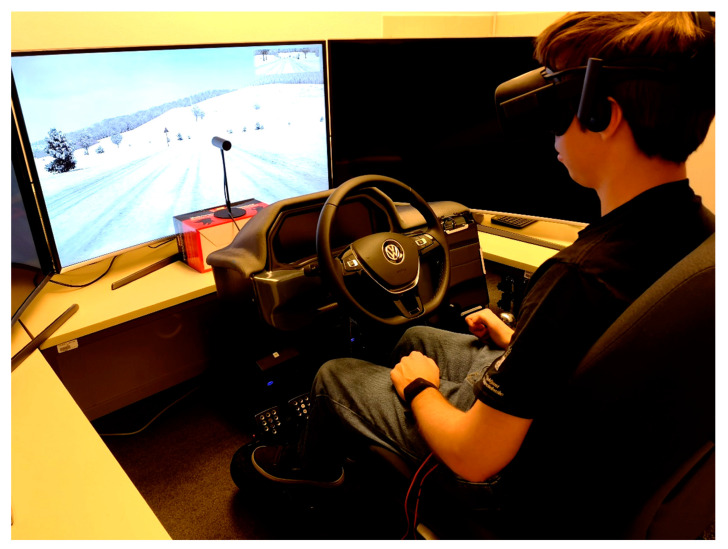
Participant prior to countryside ride in Nervtech^TM^ driving simulator at the University of Ljubljana, Faculty of Electrical Engineering. The central UHD display was used to show the view of the driver from within the VR headset to the experimenter.

Recent studies [59,60] have explored and validated the use of driving simulators as a very useful research tool for assessment of driving comfort and carsickness in AVs. Our configuration of the Nervtech^TM^ driving simulator (Nervtech d.o.o., Trzin, Slovenia [61], see Figure 1) was composed of a racing car seat, a three-pedal set with a steering wheel (Fanatech, Endor AG, Landshut, Germany) [62,63], a virtual reality (VR) headset (Oculus, Facebook Technologies LLC, Menlo Park, CA, USA) [64] and AV simulation software (AV simulation, Boulogne, France) [65]. The Nervtech^TM^ driving simulator offers a proprietary state-of-the-art 4-degrees of freedom (DOF: roll, pitch, yaw, heave) motion platform. It includes also a configurable motion system which can be adapted to realistically simulate a variety of vehicles with different dynamics (e.g., sports car, family car, SUV or heavy truck) [61]. A VR headset was used to increase the environment fidelity. Since sickness is expected to increase with the rise of automation level in vehicles, fully autonomous driving was used (SAE level 5) [66]. Credibility of measurement is also better in fully autonomous vehicles, as potential artefacts to the EGG signal by motion or steering are reduced.

**Figure 2:**
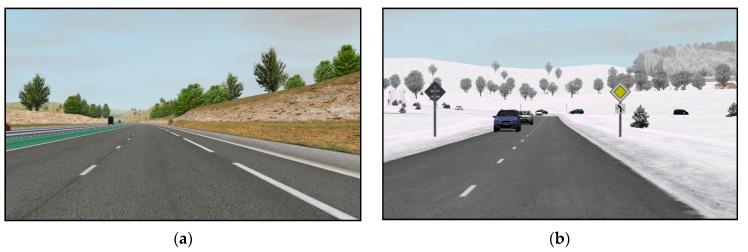
Screenshots of the driving scenarios: (**a**) highway driving with low traffic; (**b**) winding country road with many vehicles and icy roads.

Within Trial 2, participants experienced an approximately 15-min-long driving scenario with different driving scenes (see Figure 2). One half took place on a straight and less dynamic highway (7 min, part A) and the other half on a curvy and more dynamic country road (7 min, part B) with 1 min for entering and exiting the highway. Half of the participants experienced drive from A to B and the other half from B to A.

*Part A—highway driving.* On the highway, there were no drastic changes in speed and position of the vehicle (instant breaking or speeding up, instant taking-over). To make the experience engaging, other traffic, weather changes, traffic signs and billboards were added. The vehicle also made some smooth lane changes. 

*Part B—countryside driving.* On the country road, course and speed changes were introduced. The vehicle crossed many crossroads (turning left and right) and roundabouts. Safety distance was sometimes shorter than recommended. The road was icy and therefore slippery. There were also significant instant changes in speed:

- entering a village—from 90 km/h to 50 km/h,
- child running across the road—from 50 km/h to 0 km/h,
- exiting a village—from 50 km/h to 90 km/h.

### 2.3. Data Acquisition

**Figure 3:**
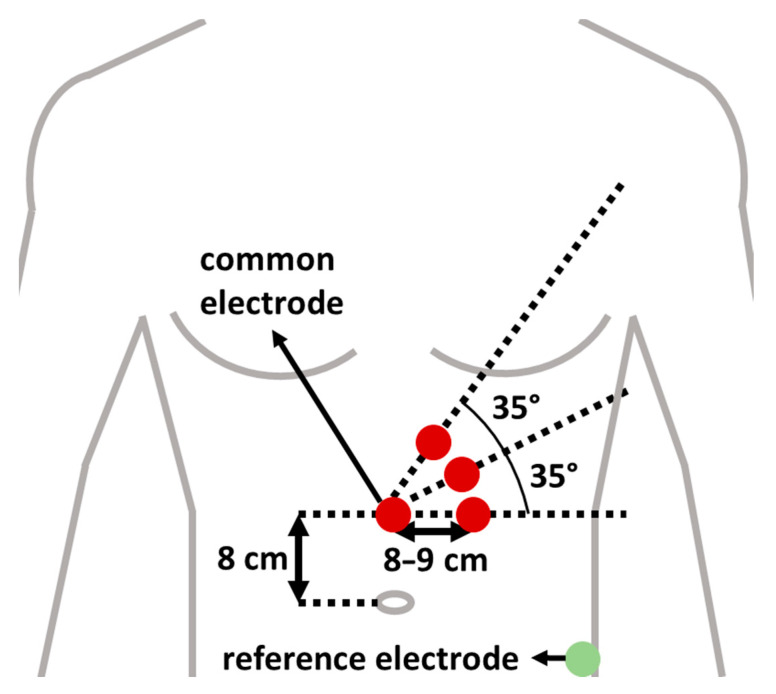
Illustration of electrode placement on body surface.

EGG was measured with a 3-channel amplification and filtering device [32] and Biopac UIM100C MP150 analog-to-digital converter (Biopac Systems, Goleta, CA, USA) [67]. Signal gain was 1000, sampling frequency 2 Hz and the resolution of A/D conversion 16 bits. The MP150 unit was connected with a cross-over Ethernet cable to the monitoring computer where real-time EGG data was stored on the hard drive. Five Ag/AgCl surface electrodes (H92SG, Kendall/Covidien, Dublin, Ireland) were placed on participant’s body for measuring 3-channel EGG. Following the recommendations of recent research [35,47,58], we prepared skin surface with abrasive gel and medical gasoline in order to decrease skin-electrode contact impedance and positioned the electrodes as demonstrated in Figure 3.

In addition to EGG signal analysis, we acquired also a set of other physiological signals to be included in the exploratory analysis of physiological responses related to MS [5]. E4 wristband (Empatica Inc., Boston, MA, USA) [68] was used for measuring galvanic skin response—GSR and heart rate—HR. The E4 was set to stream the real-time data via Bluetooth to the driving simulator where data was stored on the hard drive for post-processing. Participants were asked not to move their non-dominant hand in order to minimize the possible movement artefacts of E4’s measurements [69].

Participants were asked to continuously signal any experience of nausea or sickness by pressing a button which they held in their dominant hand. They were instructed that the more nauseated they feel, the more often should they press the button. The button was connected to the system as a simple pull-up switch to the Biopac UIM100C module and recorded alongside with EGG data on the controlling computer.

Prior to the study, participants were asked to complete a demographics and anthropometrics questionnaire, as the EGG signals are affected by the participant’s demographics (age, gender) and anthropometrics (height, weight). They were asked also to complete a questionnaire regarding their past experiences with VR. An important pitfall of simulators is also simulation sickness (SS), which is less common with high-fidelity simulators. SS exhibits similar symptoms as MS, however these tend to be less severe [18]. In order to differentiate between the two types of sicknesses we used two standardized questionnaires:

- Georgia Tech Simulator Sickness Screening Protocol Paper (GTSSSP) [21] (pre- and post- AV ride) and
- Simulator Sickness Questionnaire (SSQ) [18] (pre- and post- AV ride).

GTSSSP is used as a screening tool to identify participants that are prone to simulation sickness. With this protocol, the test participant is asked to complete the same questionnaire before and after the short test AV ride in which they rate (from 0 to 10) the perceived 17 discomfort symptoms. The pre- and post-questionnaire scores are later compared (subtracted). If any of the 17 calculated differences is greater than 5 or if three individual differences simultaneously exceed 3, the participant will not be allowed to continue [21].

Therefore, participants who the GTSSSP identified as prone to SS were not allowed to participate in the study as their results would probably be the consequence of SS instead of MS.

Furthermore, in case some of the participants would still experience SS during the study, the SSQ was used to capture this information. Participants were asked to complete SSQ before and after the ride in autonomous vehicle (Trial 2). SSQ is a commonly used questionnaire for simulation sickness assessment in VR systems. It asks participants to rate (none, slight, moderate, severe) 16 discomfort symptoms, which through some calculations provide scores of three sub-scores: nausea, oculomotor, disorientation and an overall score of SS [18]. In this study, we observed the overall SSQ score to explore whether the participants experienced SS, whereas the SSQ sub-score nausea was used as an additional reported variable of experienced nausea.

### 2.4. Participants

Twenty volunteers (two female) aged from 19 to 40 with a valid driving license and at least 1 year of driving experience were recruited for participation. They were mostly students or staff from the Faculty of Electrical Engineering, invited via a faculty mailing list. Average body mass index (BMI) of all participants was 24.6 ± 4.9.

EGG evaluates the slow wave activity and peak potentials of the gastric contractions by measuring gastric myoelectrical signals. As the digestion affects the gastric myoelectrical activity, participants were asked to feast at least 6 h and don’t drink (not even water) at least 2 h prior to the experiment [35].

Prior to participation, the experimenter described the procedure and every participant signed an informed consent. Participants were also given the option to stop the experiment at any time (e.g., if the sickness becomes too severe). For this, they were simply instructed to raise their dominant hand and the experimenter would stop the VR immediately.

The study was conducted in Ljubljana, Slovenia. It followed the Code of Ethics of the University of Ljubljana, which provides guidelines for studies involving human beings and is in accordance with the Declaration of Helsinki.

### 2.5. Tasks

In this study, a simulated vehicle with L5 autonomy was used. The participants were informed about the level of autonomy of the vehicle and that they would never have to take over control of the vehicle. Therefore, the participants’ primary task was only to observe the drive. However, there is a possibility that some participants would silently try to ignore the simulation with closed eyes while in VR or while experiencing (uncomfortable) sickness symptoms, which could lead to misleading data. To increase the participant’s situational awareness and the study validity, we introduced a simple task to ensure their engagement. The participants were given the following instructions:

“Although this is a level 5 AV, you should be aware of the driving environment and traffic around you at all times. Therefore, please count how many red vehicles will you see in the simulation, on the road or on the billboards.”

The total number of red vehicles in the simulation was completely randomized and controlled by the simulator AI traffic module. The final sum provided by the participants was therefore irrelevant and not recorded. 

### 2.6. Experiment Procedure

Each participant performed all steps listed in Table 1. Total duration of one session was a bit more than one hour.

### 2.7. Data Processing

Analysis and visualization of the data was done with MATLAB R2019a software (The MathWorks, Inc., Natick, MA, USA) [70]. For each subject, three trials were processed: (1) baseline recording, (2) recording when riding in a simulated AV using VR and (3) recording after the driving session. Additionally, the AV driving session with VR was divided into two parts: (2.1.) highway driving (part A), and (2.2.) countryside road driving (part B). Thus, parameters of five different time intervals were calculated for each subject. 

Prior to the visualization and analysis, EGG signals were (digitally) preprocessed using Butterworth 6th order zero-phase distortion band pass filter, with cut off frequencies from 1 cpm to 10 cpm (0.0167 Hz to 0.167 Hz). Additionally, all signals were visually observed by an experienced researcher. Since the amplitude of a raw EGG signal is very low (µV) there is a high probability of introducing noise or artefacts even with minimal movements. The observed motion artifacts were extracted and excluded manually. From three measured EGG channels, we chose the one with the fewest artefacts for further analysis.

Detection of amplitude onset was performed for each acquired EGG signal during the driving session (Trial 2). Due to the lack of previously published methods for detection of amplitude increase, we designed an algorithm based on the empirical knowledge. Sinusoidal nature of the signal suggested that, in order to track changes in overall amplitude, the first step should be extraction of signal envelope. We decided to use median filter with window width value equal to approximately 7 to 8 slow waves, thus 150 s (300 samples). Changes both in comparison to the baseline signal and to the VR signal were considered, so the following rule was applied: *Each envelope sample with the value more than 100% higher than the mean value of corresponding baseline signal, and more than 60% higher than the mean value of the VR signal envelope, was declared as sample with amplitude onset.*

We statistically compared the values of calculated parameters between the trials. The data had been tested for normality with Shapiro-Wilk normality test and for sphericity with Mauchly’s test of sphericity. When comparing only two trials (i.e., highway vs. country road), paired samples T-test was performed to evaluate the significance of statistical results where the differences between compared data were normally distributed, otherwise Wilcoxon Signed-Ranks Test was performed. For comparing the data of multiple trials with normal distribution, repeated measures ANOVA (RMANOVA) and (when the null hypothesis was rejected) Bonferroni post-hoc test were used. If the sphericity assumption had been violated, Greenhouse-Geisser correction was used. Where the distribution of the data was not found normal, Friedman’s non-parametric test was used and (when the null hypothesis was rejected) Wilcoxon Signed-Ranks post-hoc test with Bonferroni correction. A Spearman’s rank-order correlation was performed to determine the relationships between variables. Where not otherwise specified alpha level α = 0.05 was used.

Given the parameters, a one-way RMANOVA with 17 participants across three trials would be sensitive to effects of η^2^_p_ = 0.09 with 80% power (α = 0.05). A paired samples T-test with 17 participants would be sensitive to effects of η^2^ = 0.12 with 80% statistical power (α = 0.05). This means the study would be able to reliably detect effects greater than η^2^_p_ = 0.09 among three groups (trials 1–3) and effects greater than η^2^ = 0.12 between two groups (parts A and B).

## 3. Results

Analysis was performed on 17 out of 20 subjects. Three subjects were excluded due to poor signal quality and too often saturations in the signal (probably due to improper skin-electrode impedance). EGG signal from the upper-most electrode (see Figure 3) was most often used (15 times out of 34 recordings) as it included the fewest artefacts. GSR and HR measurements of four subjects were not obtained due to measurement device failure during AV riding. Therefore, analysis of GSR and HR was performed on 13 subjects. The final scores of GTSSSP for all participants were low enough so it was not expected for anyone to experience simulator sickness and therefore all research participants could participate in the AV simulation part of the study.

### 3.1. Analysis of before, during and after Effects

EGG, GSR and HR parameters that appear in Table 2 were measured and compared among all three trials of AV simulation (before, during and after). Although the RMS value of the EGG signal increased during the driving session and decreased again when completed, Friedman’s non-parametric test showed that the differences were not statistically significant, χ^2^(2) = 3.5, *p* = 0.17. Similarly, the MF increased during the driving session and decreased again after, however RMANOVA showed no statistically significant differences, F(2,30) = 1.331, *p* = 0.28. The same results apply for MFM, where Friedman’s test showed no statistically significant differences χ^2^(2) = 4.5, *p* = 0.11.

DF also increased during the driving session and decreased again after. When comparing with less strict alpha level of α = 0.1, RMANOVA showed that the differences are statistically significant, F(2,30) = 2.949, *p* = 0.07. Bonferroni post-hoc test showed that DF was significantly higher when riding compared to the DF acquired before the driving session (*p* = 0.07). RMANOVA test for CF revealed that CF was significantly different among the three trials, F(2,30) = 9.152, *p* = 0.001. Bonferroni post-hoc test showed that CF was significantly decreased when comparing “before” and “during” phases (*p* = 0.007) and also between “before” and “after” phases (*p* = 0.009). RMANOVA also showed that FSD values significantly vary among the trials, F(2,30) = 4.087, *p* = 0.03, however Bonferroni post-hoc test showed only slightly significant (using α = 0.1) increase between “before” and “after” phases (*p* = 0.08).

**Figure 4:**
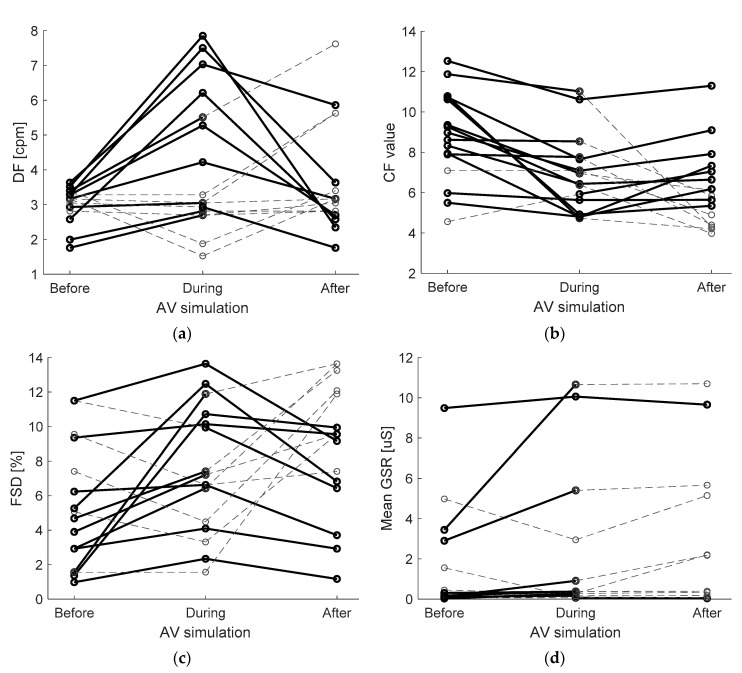
Graphical presentations of parameters that revealed a significant difference between the three trials (before, during and after the AV simulation). Dominant frequency (DF) is plotted in graph (**a**), Crest factor (CF) in graph (**b**), percentage of PSD with a value, higher than maximum magnitude of power spectrum density (MFM) divided by 4 (FSD) in graph (**c**) and mean GSR values in graph (**d**). Thick black lines indicate resulting trends (increase or decrease) that appear as expected (see Table 2). Dashed grey lines indicate trends, opposite than expected.

Friedman’s non-parametric tests showed that mean GSR value varied significantly among the trials, χ^2^(2) = 11.17, *p* = 0.004, and that the differences in standard deviation of GSR were not statistically significant, χ^2^(2) = 2.467, *p* = 0.34. Wilcox Signed-Ranks test with Bonferroni correction showed that mean GSR was significantly higher after the ride in AV than before, Z = −2.32, *p* = 0.015. Also HR mean significantly increased during the ride and decreased after, χ^2^(2) = 7.091, *p* = 0.029, however the post-hoc test showed that the differences among trials were not significant. RMANOVA for standard deviation of HR showed no significant differences in data among trials, F(2,20) = 1.548, *p* = 0.24. Graphical presentations of parameters that revealed statistically significant differences among trials can be observed in Figure 4.

### 3.2. Impact of Driving Environment

During the AV driving session (Trial 2) the participants encountered two different driving environments—a less dynamic highway and a more dynamic countryside road. While on the country road, the AV performed many lane changes, sudden brakes and take-over maneuvers compared to the highway.

**Figure 5:**
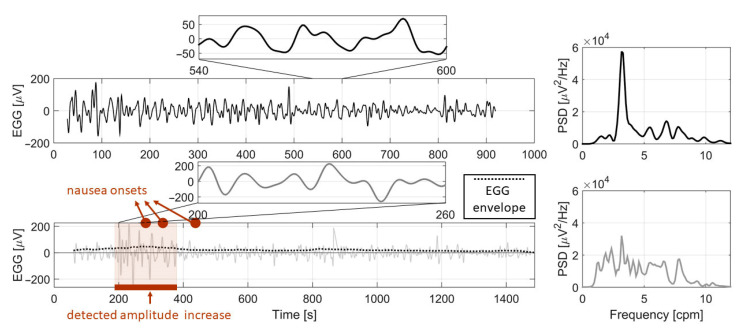
Example of resting EGG signal with zoomed part (upper left panel) and corresponding PSD of resting signal (upper right panel); example of EGG signal during AV ride with zoomed part, its envelope, marked area of detected amplitude increase and nausea onsets (lower left panel); PSD of presented EGG signal during AV ride (lower right panel). Both the detected area of amplitude increase and nausea onsets were present during the first half of AV simulation which in this case corresponds to countryside driving.

A typical EGG signal during both parts of ride is plotted in Figure 5, with marked areas of amplitude increase and nausea onsets. Plots of all participants’ EGG signals can be found in Appendix A. We statistically compared the observed EGG, GSR and HR parameters including number of nausea onsets by pressing a button.

Wilcoxon Signed-Ranks test showed that RMS and MFM were significantly higher during the countryside driving (Z = −2.58, *p* = 0.01 for RMS and Z = −2.58, *p* = 0.01 for MFM). Paired samples T-test did not show any significant difference in MF, t(16) = −0.956, *p* = 0.35, CF, t(16) = 0.642, *p* = 0.53, or FSD, t(16) = 0.303, *p* = 0.77, between the two parts. Considering the less strict alpha level of α = 0.1, paired samples T-test showed a significant decrease of DF during the countryside condition, t(16) = 1.858, *p* = 0.082. Means and standard deviations of GSR (Z = −0.784, *p* = 0.43 and Z = −0.235, *p* = 0.81) or HR (t(10) = −0.158, *p* = 0.88 and Z = −0.089, *p* = 0.93) were not significantly different between the phases. The duration of increased amplitude of EGG signal was significantly longer, t(16) = 3.001, *p* = 0.008, the increase in RMS value relative to baseline was significantly higher, Z = −2.824, *p* = 0.005, and the nausea onset button was significantly more often pressed during the countryside part.

### 3.3. Nausea Onset vs. Amplitude Increase Analysis

**Figure 6:**
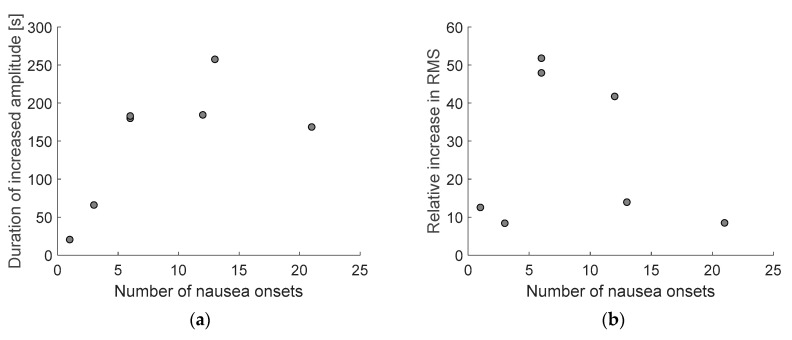
Relationship between number of nausea onsets and calculated parameters: amount of time with amplitude increase (**a**) and relative increase in RMS value (**b**).

For signals acquired during AV simulation (Trial 2) that showed both increase in amplitude and nausea onsets, two parameters (amount of time with amplitude increase and increase in RMS value relative to baseline RMS value) were calculated and compared to nausea onsets. The relationships between them are shown in Figure 6. Spearman’s rank-order correlation between the duration of increased amplitude and number of nausea onsets is r_S_(15) = 0.56, which is statistically significant with *p* = 0.02. Relative increase in RMS value is correlated with the duration of amplitude increase, r_S_(15) = 0.864, *p* < 0.001, but only moderately with the number of nausea onsets, r_S_(15) = 0.45, *p* = 0.07.

Six out of seven subjects, who reported nausea by pressing the button and had an amplitude increase, all reported nausea about one minute (71 s ± 92 s) prior to detected amplitude increase. Almost all nausea detections or onsets were detected during the countryside driving condition (Appendix A).

### 3.4. Subjective Measurements

In addition to the recorded nausea onsets in real-time using the button, all participants were asked to fill in the simulator sickness questionnaire (SSQ) to report on their subjective assessment of sickness. SSQ provides a means for quantifying overall simulation sickness and its evaluation on three sub-scales: nausea, oculomotor and disorientation. However, in the present study we only focused on nausea, since it’s correlation with motion sickness and EGG is assumed [71,72].

**Figure 7:**
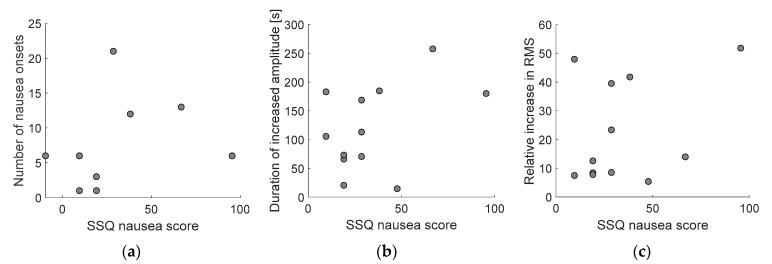
Relationships between differences in SSQ nausea score and Number of nausea onsets (**a**), Duration of increased amplitude of EGG signal (**b**) and Relative increase in RMS value of EGG signal (**c**) for subjects that reported nausea or whose EGG signal showed an increase in amplitude.

To study the consistency of subjective measures, we calculated the correlation between nausea onsets and differences in SSQ nausea score. Spearman’s correlation is r_S_(15) = 0.23, which is not statistically significant, *p* = 0.37. Relationships between differences in SSQ nausea score and number of nausea onsets are presented in Figure 7a.

Additionally, correlations between SSQ nausea score and the duration of increased amplitude and relative increase in RMS value of the EGG signal were calculated. Both the correlations of SSQ nausea with the duration of amplitude increase, r_S_(15) = 0.59, *p* = 0.01, and SSQ nausea with relative increase in RMS, r_S_(15) = 0.59, *p* = 0.01, are statistically significant. Correlations are plotted in Figure 7b,c.

## 4. Discussion

The results of the study indicate that correlations between the EGG signal parameters and self-reported sickness severity of AV riders can be established.

Considering the expected variations of parameters among the three trials, presented in Table 2, most important findings are presented in Figure 4. Regarding DF (Figure 4a), we expected alteration in frequency spectrum, but it was not clear if bradygastric or tachygastric range prevails. Since disturbances were expected in both ranges [31,32], statistical significance was unlikely to appear. Considering the most often shift to tachygastric range, slight increase of DF was expected as Tokumaru et al. suggested [50]. In line with the expectations, the DF increased during AV simulation with *p* = 0.073. Crest factor (CF) of the power spectrum density reflects the ratio of peak to effective value of the PSD, thus its decrease was expected with spectrum dispersion. As expected, the results show significantly lowered CF during the AV simulation. Therefore, we can consider EGG spectrum dispersion and gastric dysrhythmias as two potential objective indicators of nausea due to motion sickness [54].

FSD, as a measurement of PSD dispersion, also increased for each subject due to spectrum disturbances as expected. However, after the AV simulation the CF did not increase back to its original value and similarly the FSD did not decrease back to the previous value (before the AV simulation). We believe the time gap between the two series of EGG measurements (during and after the AV simulation) could have been too short and may have caused so called subject contamination. For the future studies we therefore propose a longer break between the two EGG measurement sessions or for the “after AV simulation” measurement to be longer.

Contrary to our expectations, RMS, MF and MFM did not increase significantly during the AV driving simulation. One possible reason may be a too small sample size as some F-statistics were quite high despite the lack of statistical significance. Also, since the AV driving simulation (Trial 2) consisted of two different driving environments, and the participants often felt nauseated only during the more dynamic road conditions, the overall effect may not be high enough to result in statistically significant increase of RMS, MF or MFM. Additionally, regarding MFM, it should be stated that this parameter can be decreased as a consequence of spectrum dispersion, while also increased by overall increase in signal power during AV ride, which is a bit controversial.

Multiple authors previously reported on significant changes in GSR due to thermal sweat as a symptom of MS [25,73] as well as changes of other drivers’ autonomic responses due to MS, most commonly measured with HR or HR variability (HRV) [22,23,24]. Although not being of primary interest in this study, we explored potential use of those measurements in addition to EGG. As one could expect, mean value of GSR significantly increased during the measurements, which may be associated to increased sweating as an indicator of motion sickness. The GSR measurements after the AV simulation (during Trial 3) also seem to be contaminated from the previous trials, since the values continue to increase also after the AV simulation. In contrast to our expectations, measurement of HR did not reveal any significant information.

In addition to analyzing the complete driving session during autonomous ride in VR and to confirm our assumptions and discussions in previous paragraphs we also compared two parts of AV drive with different dynamics (highway and countryside). RMS and MFM showed a significant increase during the countryside driving, where the majority of participants reported on increased nausea. Analysis of DF, CF and FSD did not reveal any significant differences between the two parts, therefore these parameters may be more interesting and expressive for overall comparisons of longer trials. Other physiological signals (GSR and HR) also did not reveal any significant changes. The number of subjectively measured nausea onsets by pressing a button was significantly higher during the more dynamic part of AV drive, complying with results of RMS and MFM. The durations and RMS values of detected amplitude increases were significantly higher during the countryside driving. These results confirm that the increases in amplitude or RMS values could be used for detection of nausea in vehicles.

Additionally, our expectations were that those increases in amplitude (both the duration and RMS) would be highly correlated with number of nausea onsets, i.e., button presses. It seems (see Figure 6) that the correlation exists only between the duration of amplitude increase and number of nausea onsets, but not between the relative increase in RMS and nausea onsets. This could probably be due to non-standardized procedure of measuring nausea by pressing a button, where every participant could have his or her own expectations and thresholds for reporting nausea and its severity. Standardization of such methodology for continuous measurements of sickness would be beneficial for any research regarding motion or simulation sickness. Nevertheless, the duration of increased amplitude appears to be a good indicator of nausea. Increased amplitude of EGG seems to appear almost simultaneously with nausea reports, indicating a promising area for further research of timings or delays between different sickness indicators. It would be of significant importance to user interface and simulator designers to know exactly when or how long after (or before) the first indication can sickness be expected.

This study included two subjective measurements, signalization of nausea onsets by pressing a button and nausea score from simulator sickness questionnaire (SSQ). SSQ was collected pre and post the driving session. It should be noted that the post SSQ was collected after the third EGG measurement session (Trial 3) in order not to interrupt the EGG recording and corrupt skin-electrode impedance contact. Consequentially the participants had about 10 min extra time to relax after being exposed to VR driving simulation and prior to questionnaire. Therefore, data collected with SSQ may be of limited significance. Although SSQ nausea score and signalized number of nausea onsets (the two subjective measurements) did not appear significantly correlated, the correlation exists between SSQ nausea score and amplitude increases in EGG signal. Both, the duration of increased amplitude and relative increase in RMS value are significantly correlated with SSQ suggesting that the proposed method of pressing the button to signalize nausea onset or detecting it though EGG amplitude increases could be two very efficient approaches. 

## 5. Conclusions

To summarize the answers to our research questions, the results of the study demonstrate that it is possible to detect increased motion sickness in a simulated autonomous vehicle by measuring EGG. Changes in the dominant frequency (DF), crest factor (CF) and percentage of power spectrum density (PSD) with a higher value than a quarter of the maximum PSD (i.e., FSD) can be used as overall indicators of sickness during AV ride. On the contrary, RMS value over a period of time and detected amplitude increases of EGG signal can be used as more accurate, short-time indicators. By applying these and the described subjective measurements, we confirmed that different driving environments also affect experienced sickness in AVs. The results showed that correlations might be established between the increased amplitude of recorded EGG (objective measures) and self-reported sickness severity by the participants (subjective measures). Participants’ self-reported nausea experiences collected with SSQ and button presses do not correlate as expected, probably due to lack of standardization of measuring sickness on a continuous level.

Longer intervals between consecutive trials or longer durations of each trial are suggested for future experiments in order to lower subject contamination from previous trials. Further improvements of the experiment may include automation of the measuring protocol and making the drivers’ experience more interactive (inclusive), probably using the in-vehicle infotainment system (IVIS).

The almost synchronous occurrences of nausea onsets and detected amplitude increases present a promising area for research regarding timing and delays among different sickness indicators. Another research area where EGG measurements could be used in future studies is semi-autonomous (conditionally autonomous) driving, where the quality of drivers’ responses to take-over requests could be subjected to MS. Therefore, more research on other options of continuously measuring sickness, time delays of individual sickness parameters and usage of EGG in semi-autonomous vehicles is required.

## Figures and Tables

**Table 1:** Experiment procedure with approximate durations, divided in three parts: introduction, experiment and post-study.

Activity	Duration (min)
Introduction	
Brief overview of: - the purpose of experiment - experiment procedure - participant’s tasks	2
Sign a consent form	1
Place EGG electrodes (to establish stable impedance) and sensors	3
Questionnaire 1: demographic data and anthropometric characteristics	2
Questionnaire 2a: Simulator Sickness Pre-questionnaire	2
Questionnaire 3a: Georgia Tech Simulator Sickness Screening Protocol Pre-questionnaire	2
Verification of the measuring system	4
Total	16
Experiment	
Baseline measurement (Trial 1)	15
Test drive in the simulated AV using VR.	5
Questionnaire 3b: Georgia Tech Simulator Sickness Screening Protocol Post-questionnaire	2
AV simulation (Trial 2)	15
Resting measurement (Trial 3)	15
Total	52
Post study	
Questionnaire 2b: Simulator Sickness Post-questionnaire	2
Detaching EGG electrodes and sensors	1
Total	3
**Total session duration**	**71 min**

**Table 2:** Expected variations of parameters, based on the listed references, and resulting trends when comparing the VR experience of autonomous driving (Trial 2) with “before” and “after” phases.

Parameter Set	Parameter	Expected Trend	Reference	Resulting Trend
EGG	RMS	Increase	[32,58]	Increase, not significant
	MF	Increase	[31]	Increase, not significant
	MFM	Increase	[57]	Increase, not significant
	DF	Increase	[31]	Increase, significant with *p* < 0.1
	CF	Decrease	[57]	Decrease, significant
	FSD	Increase	[57]	Increase, significant
GSR	Mean	Increase	[26]	Increase, significant
	St. dev.	Increase	[27]	Increase, not significant
HR	Mean	Increase	[27]	Increase, significant *
	St. dev.	Increase	[27]	Increase, not significant

* Post-hoc tests did not show any significant difference between the three trials.

**Figure A1:**
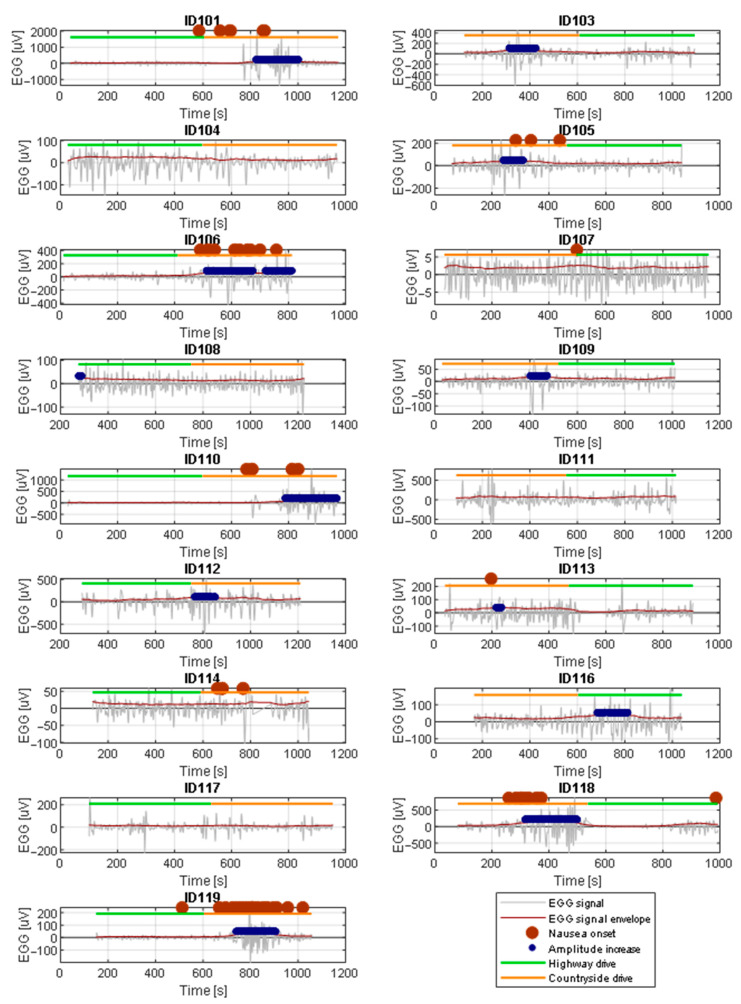
Visualization of all participants’ EGG signals during AV simulation in VR.

## Data Availability

The data presented in this study are available on request from the corresponding author.

## Appendix A

In this figure, visualization of all participants’ EGG signals during AV simulation in VR with corresponding envelope, nausea onset points, results of amplitude increase detection and marked different driving environment parts (less dynamic highway and more dynamic countryside) are presented.
